# The Dual-Specificity LAMMER Kinase Affects Stress-Response and Morphological Plasticity in Fungi

**DOI:** 10.3389/fcimb.2019.00213

**Published:** 2019-06-19

**Authors:** Joo-Yeon Lim, Hee-Moon Park

**Affiliations:** Department of Microbiology and Molecular Biology, College of Bioscience and Biotechnology, Chungnam National University, Daejeon, South Korea

**Keywords:** cell cycle, cell-wall biogenesis, cross-talk, differentiation, LAMMER kinase, morphological plasticity, stress response, virulence

## Abstract

The morphological plasticity of fungal pathogens has long been implicated in their virulence and is often influenced by extracellular factors. Complex signal transduction cascades are critical for sensing stresses imposed by external cues such as antifungal drugs, and for mediating appropriate cellular responses. Many of these signal transduction cascades are well-conserved and involve in the distinct morphogenetic processes during the life cycle of the pathogenic fungi. The dual-specificity LAMMER kinases are evolutionarily conserved across species ranging from yeasts to mammals and have multiple functions in various physiological processes; however, their functions in fungi are relatively unknown. In this review, we first describe the involvement of LAMMER kinases in cell surface changes, which often accompany alterations in growth pattern and differentiation. Then, we focus on the LAMMER kinase-dependent molecular machinery responsible for the stress responses and cell cycle regulation. Last, we discuss the possible cross-talk between LAMMER kinases and other signaling cascades, which integrates exogenous and host signals together with genetic factors to affect the morphological plasticity and virulence in fungi.

## Introduction

The family of the dual-specificity LAMMER kinases exists in all eukaryotic organisms. The presence of an “EHLAMMERILG” motif in subdomain X is a structural feature of this kinase family and is essential for phosphorylation at serine/threonine and tyrosine residues (Yun et al., 1994). The motif is virtually almost 100% identical in higher eukaryotes such as animals and plants, but not in lower eukaryotes such as fungi (Kang et al., 2013). A phylogenic analysis of the LAMMER proteins from many organisms also formed four groups: animals (group I), plants (group II), fungi (group III), and slime mold (group IV) (Duan et al., 2016). These differences can be explained by the aspects of evolution; in higher eukaryotes, members of the LAMMER kinase subfamily have evolved into proteins with different and redundant roles, but lower eukaryotes members have evolved into proteins with multiple functions or loss-of-function variants (Bender and Fink, 1994; Kang et al., 2013). LAMMER kinases are essential for viability in higher eukaryotic cells, but not in lower eukaryotic cells, meaning that there is a redundant LAMMER kinase in lower eukaryotic cells.

The LAMMER motif lying in an α-helix below the substrate-binding region (Lee et al., 1996) is important for kinase activity, substrate recognition, and subcellular localization (Savaldi-Goldstein, 2003; Kang et al., 2010). The roles for the LAMMER motif have been reported in PK12, a tobacco LAMMER kinase, with regards to two points of view. The first is that the motif is required for kinase activity, but not substrate recognition. The second is that the motif is important for its subnuclear localization (Savaldi-Goldstein, 2003). In fission yeast, however, the LAMMER motif SpLkh1 is important for kinase activity as well as substrate recognition. It also affects the distribution of SpLkh1 in the nucleus and the cell-size control and morphology (Kang et al., 2010). The differential roles of the LAMMER motif in PK12 and SpLkh1 may be explained by the fact that the variation within the less conserved LAMMER motifs in lower eukaryotes might determine substrate specificity (Kang et al., 2010).

The first member of the LAMMER kinase family reported is mouse CDC2-like kinase CLK1 (Ben-David et al., 1991; Howell et al., 1991). CLK contains highly conserved isoforms: CLK1, CLK2, CLK3, and CLK4 (Hanes et al., 1994; Nayler et al., 1997). The transcripts for all four CLK isoforms are alternatively spliced to generate mutant forms of CLK. The mutants of CLK1 form heterodimers with full-length CLK, and regulate their own splicing. These results suggest that alternative splicing processes mediated by CLK are autoregulated (Duncan et al., 1995). Moreover, the expression of CLK in stably transfected PC12 cells induces neuronal differentiation that is similar to the morphological differentiation shown by nerve growth factor treatment, indicating that LAMMER kinases are involved in signal transduction pathways. Among three human LAMMER kinases, CLK2 has been cytogenetically mapped to a region incriminated in a high percentage of spontaneous cancers such as breast cancer, and analysis of breast and prostate tumor samples demonstrates aberrantly spliced CLK2 transcripts (Talmadge et al., 1998). Therefore, it is possible that the mutation or mis-expression of LAMMER kinases, which regulate basic cellular processes, result in the initiation or progression of some cancers (Talmadge et al., 1998). The LAMMER kinase DOA (darkener of apricot) in *Drosophila melanogaster* is essential for regulation of developmental and differential processes (Yun et al., 1994); Phosphorylation of conserved splicing regulators by DOA is required for proper female sex determination (Du et al., 1998). The recessive homozygote cells are inviable, however, rare homozygotes show various defects in development of photoreceptor and imaginal discs, as well as defects in central-nerves system and segmentation patterns of embryo (Yun et al., 2000). In *Caenorhabditis elegans*, LAMMER kinase, MADD-3A, promotes muscle arm extension by ensuring that sufficient levels of EVA-1 transmembrane are displayed on the plasma membrane (D'Souza et al., 2016).

The *Arabidopsis thaliana* LAMMER kinase, AFC1, has been identified by its ability to restore Ste12-dependent function in budding yeast *Saccharomyces cerevisiae*. Subfamilies of *Arabidopsis* LAMMER kinase consist of AFC1, AFC2, and AFC3, among which only AFC1 is likely to activate the Ste12 protein. Therefore, the function of AFC1 has been specially evolved into a peculiarity, compared to that of AFC2 and AFC3 (Bender and Fink, 1994). Accumulation of gene transcripts, as well as the enzyme activity of the tobacco LAMMER kinase, PK12, are induced by the exogenous application of ethylene to tobacco leaves (Sessa et al., 1996). Heterologous expression of PK12 in *Arabidopsis* modulates the alternative splicing of mRNAs of specific developmental genes, and results in overall size reduction and prolonged life cycle (Savaldi-Goldstein et al., 2000; Savaldi-Goldstein, 2003). In rice, the two alternatively spliced transcripts of LAMMER kinase gene, OsDR11, function oppositely in the resistance against the rice pathogenic bacterium; Longer one functions negatively in disease resistance, which may suppress the Jasmonic Acid signaling, and shorter one may inhibit the function of longer one, leading to resistance against the bacterial pathogen, *Xanthomonas oryzae* (Duan et al., 2016).

**Figure 1:**
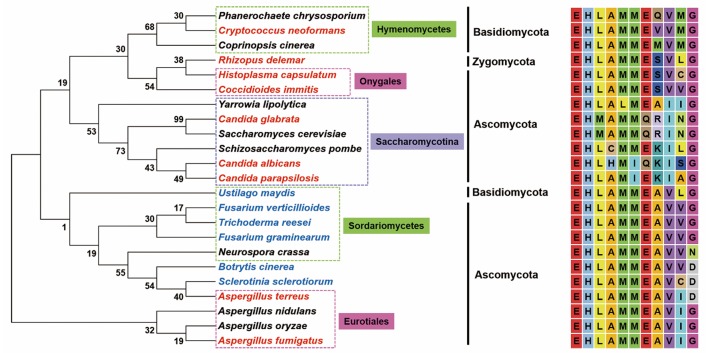
The evolutionary relationships of fungi with LAMMER motif. Neighbor-joining phylogenetic tree inferred from the amino acid sequence of LAMMER motif from 23 fungi. Bootstrap values are indicated on branches. Plant and animal pathogens are indicated in blue and red, respectively. Evolutionary analyses were conducted in MEGA7. GenBank accession number of the amino acid sequences retrieved: *Aspergillus fumigatus* (XP_753046.1), *Aspergillus nidulans* (CBF88387.1), *Aspergillus oryzae* (XP_023089190.1), *Aspergillus terreus* (XP_001214295.1), *Botrytis cinerea* (XP_024551401.1), *Candida albicans* (XP_722185.1), *Candida glabrata* (XP_445692.1), *Candida parapsilosis* (CCE42651.1), *Coccidioides immitis* (KMU82241.1), *Coprinopsis cinerea* (XP_001836332.2), *Cryptococcus neoformans* (XP_012046393.1), *Fusarium graminearum* (EYB28111.1), *Fusarium verticillioides (*XP_018750606.1), *Histoplasma capsulatum* (EEH09598.1), *Neurospora crassa* (XP_957701.3), *Phanerochaete chrysosporium* (XP_007393108.1), *Rhizopus delemar* (EIE77297.1), *Saccharomyces cerevisiae* (NP_013081.1), *Schizosaccharomyces pombe* (NP_001018187.2), *Sclerotinia sclerotiorum* (XP_001586540.1), *Trichoderma reesei* (XP_006965237.1), *Ustilago maydis* (XP_011390865.1), *Yarrowia lipolytica* (XP_501448.1).

The first LAMMER kinase family member reported in fungi is *S. cerevisiae* Kns1 (ScKns1); however, it does not show any detectable phenotypic change upon the disruption of its gene (Padmanabha et al., 1991). ScKns1 phosphorylates and interacts with mammalian splicing factors (SR proteins) *in vitro* (Lee et al., 1996). The involvement of the LAMMER kinase in the growth and morphogenesis of the fission yeast *Schizosaccharomyces pombe* was proposed based on the fact that cells with double disruption of the functional homologs of SR protein-specific kinases reveal extremely slow growth and formation of microcolonies (Tang et al., 2000). However, the first direct evidence for the *in vivo* function of LAMMER kinases in fungal species was provided by an initial characterization of SpLkh1, of which depletion induces adhesive filamentous growth and non-sexual flocculation in *S. pombe* (Kim et al., 2001). Unlike those in animals and plants, the LAMMER motifs of fungal LAMMER kinases, show similar, but not identical, amino acid sequences, reflecting the phylogenetic divergence of the kinases among fungal species (Figure 1). Since structural and mechanistical divergence of LAMMER motif has never been studied in evolutionary point of view, the biological meaning of the sequence variations cannot be easily interpreted. It is noteworthy, however, that the amino acid changes are limited within those with similar R groups. In the subclade Saccharomycotina, which exclusively contained yeast species, for example, L (Leu), M (Met), I (Ile), V (Val) are non-polar, E (Glu) and Q (Gln) are polar, and R (Arg) and K (Lys) are charged polar.

In this review, we discuss the recent findings on the biological function of fungal LAMMER kinases, particularly focusing on the molecular machinery responsible for the stress-response, cell-cycle regulation, and cross-talk with other signaling cascades, which affect the morphological plasticity and virulence in fungi (Table 1).

**Table 1:** Downstream effectors and cellular events associated with fungal LAMMER kinases^a^.

**Fungus name**	**LAMMER kinase**	**Downstream effector**	**Cellular event**	**References**
*Aspergillus nidulans*	LkhA	*brlA*	Asexual development	Kang et al., 2013
		*cnsD, ppoA*	Sexual development	Kang et al., 2013
		*stuA, nimX*	Growth and cell cycle	Kang et al., 2013
		*fksA, chsC, chsD*	Cell-wall biosynthesis	Choi et al., 2014
*Candida albicans*	Kns1	MBF-complex	Cell cycle and cell-wall biosynthesis	Lim et al., 2019
		?	Virulence	Park and Park, 2011a
*Saccharomyces cerevisiae*	Kns1	?	Adhesive filamentous growth	Park et al., 2011
		*FLO8*	Temperature sensitivity	Park and Park, 2011b
		Rpc53	Ribosome and tRNA biogenesis	Lee et al., 2012
		Cbk1	tRNA biogenesis	Sanchez-casalongue et al., 2015
*Schizosaccharomyces pombe*	Lkh1	Tup11, Tup12	Glucose repression and flocculation	Kim et al., 2001; Kang et al., 2010
		Csx1	Oxidative-stress response	Park et al., 2003; Kang et al., 2007
		Prk1	Flocculation	Park et al., 2018
		Rum1	Cell cycle and sexual differentiation	Yu et al., 2013
		?	Pre-mRNA splicing	Tang et al., 2011
		?	Protein secretion	Cho et al., 2010
*Ustilago maydis*	Lkh1	?	Cell cycle and sexual differentiation	de Sena-Tomás et al., 2015

a *Downstream effectors (genes or proteins) and cellular events presented are experimentally determined*.

## Involvement in Cell Growth and Cell-Wall Biogenesis

In fission yeast *S. pombe*, the LAMMER kinase deletion mutant (Splkh1Δ) is viable, grows a little bit slowly than its wild-type counterpart, and exhibits non-sexual flocculation in both nutrient-rich and minimal liquid media (Kim et al., 2001); Flocculation requires galactose residues of the cell wall glycoprotein, which may serve as receptors of divalent cation-dependent non-sexual flocculation. The Splkh1Δ cells show adhesive growth, which makes them stick to the agar surface even after the colony washing with tap water. The morphology of adhesive cells is pseudohyphal and filamentous; however, the cells do not invade into the agar media, as reported in case of *S. cerevisiae* (Gimeno et al., 1992). Overexpression of *ScKNS1* or *lkh1*^+^ does not affect the morphology of *S. pombe* in liquid culture; however, the *ScKNS1* reverses the non-sexual flocculation of Splkh1Δ, indicating that the function of the LAMMER kinase in *S. pombe* can be substituted partially by the *S. cerevisiae* LAMMER kinase, which may function in different ways in *S. cerevisiae* (Kim et al., 2001). The haploid cells of *ScKNS1*-deletion mutants (Sckns1Δ) with S288c-background show no noticeable phenotypic changes (Liu et al., 1996). However, both haploid and diploid cells of Sckns1Δ with the Σ1278b-background, with which one can induce filamentous and adhesive growth in contrast to those with the S288c-background (Gimeno et al., 1992), show defects with regards to filamentous growth under filamentous growth-inducing conditions, such as nitrogen starvation and butanol treatment, suggesting the possible cross-talk between the Flo8 of the PKA signal transduction pathway (Liu et al., 1996) and LAMMER kinase pathways in transducing a signal for regulating filamentous growth.

In the human opportunistic pathogen *Candida albicans*, LAMMER kinase deletion mutant (Cakns1Δ) cells show hyphal defects only on solid hypha-inducing medium (Lim et al., 2019); The Cakns1Δ cells produce colonies with wrinkled center (consisting of yeast, pseudohyphae, and hyphae) and few peripheral filaments on solid media. Similar to the Splkh1Δ cells, the Cakns1Δ cells show adhesive growth on agar surface and flocculation in submerged culture (Lim et al., 2019). In the filamentous fungus *A. nidulans*, LAMMER kinase is involved in vegetative growth and polarity determination of the hyphae (Kang et al., 2013); The LAMMER kinase deletion (AnlkhAΔ) cells are viable, but show the increase in the proportion of bipolar or multipolar germlings, indicating changes in polarity during germ-tube formation. The hyphae from the colony margin of the AnlkhAΔ strain show hyper-branching in contrast to those of the wild type, which show apical polarity during hyphal extension and moderate-branching. The radial growth of AnlkhAΔ strain is reduced, but the density of the mycelial balls produced in submerged culture is higher than that of the wild type, possibly due to the delay in the disintegration (autolysis) of the mycelia (Kang et al., 2013).

Non-sexual flocculation in *S. pombe* is induced by the deletion of factors, which negatively regulates the expression of cell-surface flocculins, such as Prk1 (Watson and Davey, 1998), transcriptional repressors Tup11 and 12 (Kang et al., 2010), and the ribosomal protein Rlp32 (Li et al., 2013; Liu et al., 2015), and by the overexpression of some adhesins (Matsuzawa et al., 2011). The flocculating activity of the Splkh1Δ mutant (Kim et al., 2001) is increased by the additional deletion of the *prk1*^+^ gene, but is nullified by the overexpression of Prk1 (Park et al., 2018). Consistent with the *prk1*^+^-deletion and -overexpression experiments, the transcriptional level of *prk1*^+^ is also significantly decreased in the Splkh1Δ mutant, indicating that Prk1 functions at the downstream pathway of SpLkh1-mediated non-sexual flocculation. In addition, non-sexual flocculation of the *prk1*^+^-deletion mutant cells is galactose-specific and divalent-cation-dependent (Park et al., 2018), which is similar to the case with the Splkh1Δ mutant (Kim et al., 2001). The flocculation in submerged culture and filamentous growth on agar surface shown by the Splkh1Δ mutant appear to result from changes of cell wall proteins in *S. pombe*; Polyacrylamide gel electrophoresis and subsequent identification of differentially expressed extracellular proteins from wild-type and Splkh1Δ mutant cells by tandem mass spectrometry reveal the upregulation of the glycolipid-anchored surface precursor β-glucosidase, the cell surface 1,3-β-glucosidase, and exo-1,3 β-glucanase in the Splkh1Δ mutant (Cho et al., 2010). In *C. albicans*, the LAMMER kinase controls normal yeast growth by negatively regulating hypha-specific genes such as *HYR1, ECE1*, and *ALS3*. Up-regulation of these genes in the Cakns1Δ cells indicates that the LAMMER kinase controls normal yeast growth by negatively regulating hypha-specific genes (Lim et al., 2019).

In *A. nidulans*, AnLkhA regulates the expression of the *fksA* gene encoding β-1,3-glucan synthase and *chsC* and *chsD* gene encoding chitin synthase in different ways. The *fksA* is up-regulated by AnLkhA during vegetative growth before the acquisition of developmental competence. However, *chsC* is down-regulated, but *chsD* is up-regulated by AnLkhA during vegetative growth after the acquisition of developmental competence (Choi et al., 2014). It is noteworthy that *chsC* and *chsD* play different roles in growth and development; *chsC* plays a role in maintaining integrity of vegetative hyphal wall and developing sexual structures such as conidiophores (Fujiwara et al., 2000), whereas *chsD* is required for vegetative growth and development (Specht et al., 1996). Consistent with the cell wall-related gene expression, the amount of β-1,3-glucan is decreased in the AnlkhAΔ strain, compared to its wild-type counterpart, but the amount of chitin is increased (Choi et al., 2014). In yeast, the binding of cell wall-perturbing agents to sensors on the cell surface triggers the CWI (Cell Wall Integrity) and HOG signaling pathways (García-Rodriguez et al., 2000), and therefore, synthesis of cell-wall β-1,3-glucan (Roemer et al., 1994) and chitin (Igual et al., 1996) is modulated at a transcriptional level. Sensitivity to cell wall-perturbing agents is indicative of the cell-wall composition; Cacofluore White (CFW) specifically binds to the chitin, whereas Congo Red (CR) binds to the chitin and β-glucan and inhibits chitin synthases (Roncero and Durán, 1985; Kovács et al., 2013). In *C. albicans*, strains with single or double deletion of the *CaKNS1*, are hypersensitive to the treatment of CFW and CR, and increase predominant chitin deposition around the bud neck of yeast form and in the septa of the filamentous form (Lim et al., 2019). Interestingly, the expression of *CHS* genes for chitin synthase shows no significant changes, however, the expression of the *FKS1* gene for 1,3-glucan synthase is decreased by the treatment of CFW (Lim et al., 2019). In *A. nidulans*, AnlkhAΔ strain shows no significant difference with regards to the sensitivity against CFW and CR (Choi et al., 2014). Antifungal drug tests, on the other hand, the AnlkhAΔ strain reveals resistance to Nikkomycin Z, a specific inhibitor of the ChsC (Park et al., 2004), but not to Terbinafine, a specific inhibitor of ergosterol synthesis, and thus, defects in cell-wall biosynthesis (Choi et al., 2014).

## Stress-Response, Cell Division, and Differentiation

The biological effects of ROS are controlled by a wide spectrum of enzymatic defense mechanisms (Guemouri et al., 1991). Wild-type and Splkh1Δ mutant cells of *S. pombe* do not differ with regards to their response to menadione-mediated oxidative stress; however, Splkh1Δ cells are more sensitive to H_2_O_2_-mediated oxidative stress and show dramatic decrease in catalase (CAT) activity relative to that of wild-type cells. The expression of the gene for CAT, *ctt1*^+^, is not completely abolished, but its mRNA is more rapidly disappeared than that in case of the wild-type cells (Park et al., 2003). In *S. pombe*, the Spc1/Sty1 MAP kinase cascade predominantly transduces stress signals, activating transcription factors such as Atf1 or Pap1, which then induce transcription of anti-oxidant genes such as *ctt1*^+^ under oxidative stress conditions (Degols and Russell, 1997). Atf1 is activated by a broad concentration range of H_2_O_2_, whereas Pap1 is activated by the low levels of H_2_O_2_ (Quinn et al., 2002). The expression of *pap1*^+^ in response to H_2_O_2_-mediated oxidative stress is not affected but that of *atf1*^+^is affected by the deletion of SpLkh1, indicating that the SpLkh1-dependent, oxidative stress-induced expression of *ctt1*^+^ is mediated by Atf1, not by Pap1 (Park et al., 2003). Additionally, among the genes whose expression is dependent on Atf1, the expression of the *sod1*^+^ gene, which encodes the Cu and Zn-containing superoxide dismutase (CuZnSOD), is also greatly affected by SpLkh1, suggesting that the SpLkh1 affects not only the expression of *ctt1*^+^, but also the expression of other Atf1^+^-dependent genes in response to oxidative stress (Park et al., 2003). Interestingly, in Splkh1Δ mutant cells, the reduction in *atf1*^+^ mRNA upon H_2_O_2_ treatment is not related to the expression of the components of the Spc1 MAPK pathway, suggesting possible involvement of other factor(s) in the expression of *atf1*^+^ under oxidative stress conditions (Kang et al., 2007). Different from the Splkh1Δ mutant cells, the Cakns1Δ mutant strains are not sensitive to oxidative stresses, suggesting that the CaKns1 may not be linked to the signaling pathway for oxidative-stress response in *C. albicans* (Lim et al., 2019).

The RNA-binding Csx1 protein is phosphorylated by the Spc1 MAP kinase and binds directly to *atf1*^+^ mRNA, which in turn stabilizes the *atf1*^+^ mRNA and thus maintains normal levels of Atf1 under oxidative stress conditions (Rodriguez-Gabriel et al., 2003). However, the Spc1-dependent phosphorylation of Csx1 is not sufficient for its function and various combinations of the mutation in Spc1-dependent phosphorylation sites show no apparent effect on Csx1 function under oxidative stress, suggesting participation of other kinase(s) for the Csx1 function under oxidative stress conditions (Rodriguez-Gabriel et al., 2003). SpLkh1 is also involved in activation of Csx1 function in response to oxidative stress; SpLkh1 shows *in vivo* interaction with Csx1, which is increased by H_2_O_2_ treatment response, but not by non-oxidative stress. The reduced binding of Csx1 to *atf1*^+^ mRNA results in the reduction of *atf1*^+^ mRNA levels in Splkh1Δ cells under oxidative stress conditions, which in turn renders the mutant cells sensitive to oxidative stress conditions. The binding of Csx1 to *atf1*^+^ mRNA is activated by both Lkh1and Spc1, however, the interaction between Lkh1 and Csx1 upon oxidative stress is not abolished by deletion of *spc1*^+^ (Kang et al., 2007). Although the Spc1 responds to a broad concentration range of H_2_O_2_ and SpLkh1 responds to low levels of H_2_O_2_, the activation of Csx1 under oxidative stress conditions may be mediated by the concerted action of the SpLkh1 and Spc1 pathways (Park et al., 2003; Kang et al., 2007).

The budding yeast *S. cerevisiae* Σ1278b strain, which shows haploid and diploid filamentous growth, is heat sensitive at 37°C, which is a mild heat stress condition, compared to the S288c strain. The heat sensitivity of the Σ1278b strain is suppressed by the deletion of ScKns1 and the addition of sorbitol into the medium, suggesting that the Flo8 of PKA pathway and ScKns1 may interact to transduce a signal for heat-stress response (Park and Park, 2011b). It is also noteworthy that the defects of the Sckns1Δ with regards to filamentous and adhesive growth are suppressed by the overexpression of each gene encoding the components of the MAPK signaling pathway, such as *STE11, STE12*, and *TEC1*, but not by the overexpression of each gene encoding the upstream components, *RAS2* and *STE20* (Park et al., 2011). These results indicate that the LAMMER kinase may act between the Ste20 and the Ste11 of MAPK signaling pathway and also suggest the possibility that the LAMMER kinase may mediate cross talk between PKA and MAPK pathways in *S. cerevisiae*.

In *S. pombe*, Tup11 and Tup12 regulate the responses to stresses such as salt stress, heat shock, and oxidative stress at transcriptional level (Fagerstrom-Billai and Wright, 2005). The double deletion of *tup11* and *tup12* reveals non-sexual flocculation and defect in mating and stress response (Janoo et al., 2001; Fagerstrom-Billai and Wright, 2005), which mirror those of the Splkh1Δ mutant (Kim et al., 2001). The Tup11 and Tup12 are phosphorylated by SpLkh1 *in vitro* and *in vivo*. The overexpression of Tup11 and Tup12 enhance the non-sexual flocculation and adhesive growth of the Splkh1Δ mutant and wild-type cells. While the flocculation phenotype of Splkh1Δ cells is reversed by the expression of Tup11 and Tup12 with the co-repressor Ssn6, the flocculation phenotype of the Δ*tup11*Δ*tup12* mutant is not reversed by the introduction of *lkh1*^+^; this indicates that the expression of the genes related to the non-sexual flocculation is repressed by Tup11 and Tup12, which are activated by the SpLkh1-dependent phosphorylation. However, the involvement of additional factor(s) other than Lkh1 could not be excluded, because the phosphorylation of Tup-repressor for their activation may not be completely dependent on SpLkh1 (Kang et al., 2010).

The eukaryotic cell cycle is regulated, in the G1 phase before the transition into the S-phase, and in the G2 phase before the entry into M phase, by the action of cyclin-dependent kinases (CDKs) (Stern and Nurse, 1996). In fission yeast, one of the biochemical events that regulates the activity of the only CDK, Cdc2, is the modulation by the CDK inhibitor Rum1 (replication uncoupled from mitosis). Rum1 blocks the cell cycle progression in the G1 phase, allowing the initiation of meiotic cycle in response to environmental conditions such as nitrogen starvation (Daga et al., 2003). The Splkh1Δ cells display delayed growth and shorter size, and lower cell numbers with 2C DNA content, compared to the case for the wild-type cells, suggesting that SpLkh1 controls cell cycle progression in G1 phase, and thus affects the cell size (Park et al., 2003). Additionally, the SpLkh1 is expressed in a cell cycle-dependent manner and shows its maximum expression during mitosis and cytokinesis (Tang et al., 2011), supporting the suggestion on the involvement of SpLkh1 in the cell-cycle regulation. The Splkh1Δ mutant cells pass through the G1/S transition faster than their wild-type counterparts. The SpLkh1 also interacts with and phosphorylates Rum1, indicating that phosphorylation of Rum1 by SpLkh1 is required for the activation the CDK inhibitor Rum1 (Yu et al., 2013). This indication is contrasting with results from previous reports, which showed negative effects of phosphorylation on Rum1 function, such as the triggering of Rum1 degradation and the inhibition of the CDK inhibitor activity of Rum1 (Benito et al., 1998). In *C. albicans*, CaKns1 also plays a positive role in the expression of cell cycle-related genes that are regulated by the MCB (*Mlu* I-cell cycle box) –binding factor (MBF) complex, which regulates the G1 phase to S phase progression in *S. cerevisiae* and *S. pombe*, under conditions of DNA replicative stress such as methyl methanesulfonate (MMS) treatment (Lim et al., 2019). Involvement of AnLkhA in the regulation of nuclear-division cycle is indicated by the defects in septum formation and nuclear behavior not only in the vegetative hyphae, but also in conidiophore stalks in the AnlkhAΔ mutant strain, due to the defect in the transcription (and thus, translation) of the Cdc2 homolog NimX (Kang et al., 2013). In *Ustilago maydis*, the LAMMER kinase mutant (lkh1(Q488^*^)) is highly sensitive to the treatment of DNA-damaging agents, such as UV, MMS, and hydroxyurea (HU). In contrast, the LAMMER kinase deletion mutant (Umlkh1Δ) has a less severe phenotype. The lkh1(Q488^*^) and Umlkh1Δ mutants exhibit reduced heteroallelic recombination and aberrant chromosome segregation, suggesting UmLkh1 functions with regards to cell-cycle regulation, and sexual differentiation (de Sena-Tomás et al., 2015).

Unlike the *rum1* deletion, the *Splkh1* deletion does not completely block entry into the meiotic cycle (sexual differentiation) in response to nitrogen starvation, indicating that SpLkh1 is not the only factor for Rum1 activation (Yu et al., 2013). It is noteworthy that the *csx1* deletion also results in defects with regards to sexual differentiation; the *csx1*-deleted mutant cells are partially sterile, due to a reduced amount of *ste11*^+^ mRNA. The Ste11 regulates the transcription of many genes for the initial steps of conjugation and meiosis during sexual differentiation of *S. pombe* (Matia-Gonzalez et al., 2012). These results indicate that the function of SpLkh1 in sexual differentiation is mediated not only via Rum1, but also via Ste11. These results may provide an answer to the question raised previously about the possible coordination of sexual differentiation and oxidative-stress response, as well as the role of RNA-binding proteins in the adaptation of cells to environmental signals (Matia-Gonzalez et al., 2012). These results also suggest that the SpLkh1 may mediate the interplay sexual differentiation and oxidative-stress response.

AnLkhA modulates the conidiophore morphogenesis *via* unidentified upstream factor(s) of the central pathway for asexual differentiation, and the development of sexual organs for ascospore production by influencing the transcription of the components of the COP9 signalosome (CNS) (Kang et al., 2013). The pleiotropic phenotypes of the AnlkhAΔ strain could be supported by the association of CNS with molecular events of developmental processes in animals, such as transcriptional regulation, protein phosphorylation, DNA-damage response, and the cell cycle (Kato and Yoneda-Kato, 2009). AnLkhA also modulates the transcription of a developmental gene, *stuA*, which is associated with many aspects of fungal differentiation, such as sporogenesis, pathogenicity, and the production of secondary metabolites (Ohara and Tsuge, 2004; Tong et al., 2007; García-Pedrajas et al., 2010; IpCho et al., 2010). It is noteworthy that the nucleotide sequence of StuA response elements (StREs) is identical to that of the MCB-binding motif and that the DNA-binding motif of StREs is enriched with promoter region of genes belonging to the functional MIPS category “Cell cycle and DNA processing” in ascomycetous fungi including *S. cerevisiae* (Lysøe et al., 2011). Sequence analysis of the *nimX* and *fksA* promoter region also reveals two StREs (Kang et al., 2013; Park et al., 2014). It is also noteworthy that the recombinant StuA proteins bind to PCR-amplified fragments from the *fksA* promoter (Park et al., 2014). These results suggest that the transcription of genes for cell-division cycle (e.g., *nimX*) and cell-wall biogenesis (e.g., *fksA*) is modulated by StuA, and thus, that AnLkhA modulates the regulatory circuit(s), which governs cell cycle and cell-wall biogenesis in conjunction with differentiation in *A. nidulans*.

## Morphogenetic Plasticity and Virulence

In most fungal pathogens, the cell wall is one of the key virulence factors and is essential for cell viability, morphology, and stress response. Host cells interact with fungal cell wall components, such as glycosylphosphatidylinositol (GPI)-anchored proteins or polysaccharides. The main carbohydrates of the cell wall, chitin and glucan, are well-known targets of antifungal drugs. Therefore, numerous studies have examined cell wall biogenesis in relation to pathogenesis (Lenardon et al., 2010). Yet, the role of LAMMER kinases in fungal virulence has not yet been examined precisely. LAMMER kinases play pivotal roles in cell-wall biogenesis, stress response, cell cycle, and thus, morphogenetic plasticity, which is intimately coupled with virulence in pathogenic fungi.

**Figure 2:**
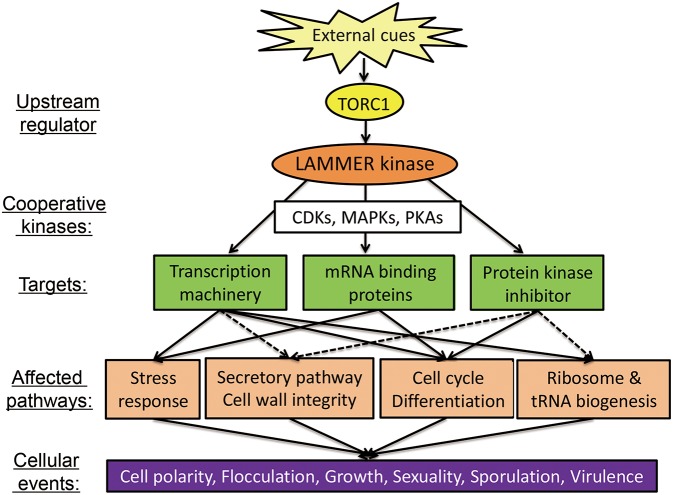
Biological functions of the LAMMER kinase in fungi. Scheme shows the biological processes regulated by LAMMER kinase. Protein kinases and protein kinase pathways linked to the LAMMER kinase-dependent processes are also depicted. CDK, cyclin-dependent kinase; MAPK, mitogen-activated protein kinase; PKA, protein kinase A; TORC, target of rapamycin complex.

In *C*. *albicans*, the Cakns1Δ cells are not sensitive to oxidative stress, osmotic stress, and ER stress, but are hypersensitive to CFW and CR; Deletion of *CaKNS1* causes changes in the cell wall composition, which lead to defects in the CWI signaling pathway, and thus, induce the expression of genes encoding GPI-anchored cell-wall proteins, which are known to be expressed at high levels following β-1,3 glucan inhibitor and CFW treatment (Lim et al., 2019). In virulence tested using a mouse model, the mean survival time for the Cakns1Δ strains are longer than that in case of the wild-type strain and the fungal burden on the kidneys for the Cakns1Δ strains decreases, compared to that for the wild-type strain (Park and Park, 2011a). The test with the mouse model, however, needs to be reevaluated to draw a conclusive answer, because the wild-type strain used in the virulence test is not congenic to deletion strains. In the test for the adhesive ability of *C. albicans* cells *in vivo* using a zebrafish egg infection model system, the Cakns1Δ cells produce a thick mycelial layer with dense hyphae on the embryonal surface. However, there is no significant difference between the hatching rate of the zebrafish eggs infected with the congenic wild-type and deletion strains (Lim et al., 2019). Although LAMMER kinase does not act as a virulence factor, it affects fungal virulence in a multiphasic manner. It plays roles in modulating multiple aspect of cellular processes such as dimorphic switch, oxidative-stress response, sporulation, and cell cycle in response to the environmental cues including host signals (Figure 2). In order to understand the role of LAMMER kinases in fungal virulence together with morphogenetic plasticity, studies with other fungal pathogens including *Cryptococcus neoformans, Aspergillus fumigatus*, and *Magnaporthe grisea* are required.

## Conclusion and Future Perspectives

Dual-specificity LAMMER kinases have been reported to be conserved across species ranging from yeasts to animals, and have multiple functions. The LAMMER kinases of higher eukaryotes (i.e., Clk/Sty family in mammalian cells, *Drosophila* DOA, and tobacco PK12) regulate the splicing of pre-mRNA via phosphorylation and interactions with serine/arginine-rich splicing factors. As summarized and depicted in Table 1 and Figure 2, LAMMER kinase plays a pivotal role in diverse aspects of fungal development including pathogenicity. In yeasts, LAMMER kinases are involved in filamentous growth, asexual flocculation, stress responses, and the cell cycle by regulating the transcription of genes and translation, as well as the post-translational modification of the proteins. In filamentous fungi, LAMMER kinases affect vegetative growth, asexual and sexual development, cell-wall biogenesis, and DNA-damage response by regulating the expression of developmental genes and cell cycle-related genes. With regards to the virulence of the pathogenic fungi, LAMMER kinases modulate dimorphic transition, cell-wall integrity, adherence to the host cell surface, and cell-cycle regulation. Fungal LAMMER kinases are also involved in pre-mRNA splicing, as in case of higher eukaryotes. Thus, novel information from the study on the function and regulatory mechanism of LAMMER kinases in fungi will offer insights into the conserved biological roles of dual-specificity LAMMER kinases in higher eukaryotes.

For the better understanding of the function of LAMMER kinases *in vivo*, many issues are yet to be addressed. In particular, molecular mechanisms ranging from those underlying the recognition of external cues such as oxidative stress and/or nutrient deprivation to those underlying morphogenetic changes including vegetative growth, and asexual and sexual development, should be elucidated.

Further investigation of the networks of kinase and phosphatase cascades, which couple growth to cell-wall biogenesis and the cell cycle, are also required. To this end, attention on recent reports mentioned below may be required.

Lee et al. (2012) reported the cross-talk between target of rapamycin (TOR)-dependent signaling and LAMMER kinase in *S. cerevisiae*; The ScKns1 is differentially expressed and hyper-phosphorylated, and accumulates in the nucleus upon rapamycin treatment. In cooperation with a specific GSK-3 family member, Mck1, ScKns1 finally modulates the growth-promoting activity of RNA polymerase III (Pol III) transcription. A link between TOR Complex 1 (TORC1) activity, ScKns1 phosphorylation of the β regulatory subunit of CK2 (Ckb1), and CK2 regulation of pol III transcription has also been reported; The ScKns1-dependent phosphorylation of Ckb1 correlates with the reduced occupancy of Ckb1 on tRNA genes after rapamycin treatment, which in turn is likely to reduce its activation of the poly III transcription (Sanchez-casalongue et al., 2015). Recent study on the function of the conserved Greatwall-Endosulfine regulatory (GER) pathway shows an interplay between cell growth and cell-cycle machinery; Activation of GER pathway upon TORC1 inhibition regulates the cell-cycle progression at the G1 phase in budding yeast, and at the G2/M phase transition in fission yeast, and the entry into quiescence in both budding and fission yeasts (Pérez-Hidalgo and Moreno, 2017).

## Author Contributions

J-YL: collection of data, writing the manuscript, and figure drawing. H-MP: study plan, collection of data, writing the manuscript, and manuscript revision.

### Conflict of Interest Statement

The authors declare that the research was conducted in the absence of any commercial or financial relationships that could be construed as a potential conflict of interest.
